# Sphingolipidomic Profiling of Peripheral Blood Mononuclear Cells Reveals a Distinct Immunometabolic Signature Across Patients with Essential Obesity and Metabolic Syndrome Compared to Normal-Weight Healthy Subjects

**DOI:** 10.3390/jcm15103634

**Published:** 2026-05-09

**Authors:** Antonello E. Rigamonti, Michele Dei Cas, Paola Signorelli, Adele Bondesan, Diana Caroli, Silvano G. Cella, Alessandro Sartorio

**Affiliations:** 1 Department of Clinical Sciences and Community Health, Dipartimento di Eccellenza 2023–2027, University of Milan, 20129 Milan, Italy; silvano.cella@unimi.it; 2 Department of Health Sciences, University of Milan, 20142 Milan, Italy; m.deicas@unimi.it (M.D.C.); paola.signorelli@unimi.it (P.S.); 3 Experimental Laboratory for Auxo-Endocrinological Research, Istituto Auxologico Italiano, IRCCS (Istituto di Ricovero e Cura a Carattere Scientifico), 28824 Piancavallo-Verbania, Italy; a.bondesan@auxologico.it (A.B.); d.caroli@auxologico.it (D.C.); sartorio@auxologico.it (A.S.)

**Keywords:** essential obesity, metabolic syndrome, sphingolipids, peripheral blood mononuclear cells, IDF diagnostic criteria

## Abstract

**Background**: While sphingolipid alterations in obesity and metabolic syndrome (MS) have been extensively studied in plasma, their intracellular regulation within immune cells remains poorly characterized. Here, we introduce a PBMC-based sphingolipidomic approach that provides a novel immunometabolic perspective beyond traditional analyses of circulating lipids. **Methods**: Targeted sphingolipidomics was performed in peripheral blood mononuclear cells (PBMCs) from normal-weight healthy (NWH) subjects, and patients with essential obesity (EO) or MS. Multivariate integration (principal component analysis [PCA], hierarchical clustering, and partial least squares discriminant analysis [PLS-DA]) was combined with selected univariate models to explore lipid patterns and associations with cardiometabolic variables. **Results**: PBMC profiling identified a selective intracellular sphingolipid signature, with 10 species significantly altered across groups (FDR < 0.05), including ceramides, dihydroceramides, glycosphingolipids, and ceramide ratio indices. PCA showed that the first two components explained ~72% of total variance, with PC2 driving group separation. EO and MS displayed partial overlap, consistent with a shared metabolic phenotype, while both differed from NWH. Multivariate models highlighted ceramide ratios (e.g., CER16/24, and CER18/24) as key discriminators. Associations with cardiometabolic variables were limited and modest (adjusted R^2^ ≈ 0.06–0.09), indicating that lipid alterations reflect integrated metabolic dysregulation rather than single clinical drivers. **Conclusions**: PBMC-based sphingolipidomics reveals a distinct intracellular immunometabolic remodeling in EO and MS, capturing aspects not detectable in plasma. These findings support the relevance of immune cell lipid profiling as a potential source of integrative biomarkers and provide insight into immune–metabolic crosstalk underlying metabolic disease.

## 1. Introduction

Essential obesity (EO) and metabolic syndrome (MS) represent major global health challenges, characterized by complex interactions between metabolic dysregulation, chronic low-grade inflammation, and immune system activation [1,2]. These conditions are strongly associated with increased risk of cardiovascular disease, type 2 diabetes (T2D), and mortality [3,4]. Despite extensive research, the molecular mechanisms linking excess adiposity to systemic metabolic dysfunction remain incompletely understood [5].

Among lipid classes, sphingolipids have emerged as key regulators of metabolic homeostasis [6,7]. Ceramides (Cers), the central “biochemical hub” of sphingolipid metabolism, have been implicated in insulin resistance through inhibition of Akt signaling, mitochondrial dysfunction, and promotion of apoptosis [8,9]. Downstream metabolites, including sphingomyelins (SMs) and glycosphingolipids such as hexosylceramides (HexCers), lactosylceramides (LacCers), and gangliosides (GMs), contribute to membrane organization, receptor signaling, and inflammatory responses [10,11]. Moreover, intermediates of de novo synthesis, such as dihydroceramides (DHCers), are increasingly recognized as early indicators of metabolic stress [12]. Collectively, these lipid classes suggest a coordinated remodeling of sphingolipid metabolism in metabolic imbalance, with potential differences in pathway activation between EO and the more advanced MS phenotype.

Most studies investigating sphingolipid alterations in EO and MS have been conducted in plasma or serum [13]. While informative, circulating lipid levels primarily reflect systemic lipid transport and inter-organ fluxes and may not fully capture intracellular metabolic processes [14]. This represents a key knowledge gap, as intracellular lipid remodeling—particularly in immune cells—may directly link metabolic stress to inflammation. Peripheral blood mononuclear cells (PBMCs) constitute a biologically active compartment involved in immune responses and inflammatory signaling [15]. In obesity and MS, PBMCs undergo functional and metabolic reprogramming, including shifts in substrate utilization and inflammatory activation [16]. Therefore, PBMC-based sphingolipidomics offers a complementary and potentially more mechanistically informative perspective compared to circulating lipid analyses.

Although intracellular lipid profiling in immune cells remains relatively limited, emerging studies have begun to explore lipidomic changes in PBMCs and related immune populations, highlighting their relevance for understanding immunometabolic interactions [15,16]. However, a comprehensive characterization of sphingolipid metabolism in PBMCs across different stages of metabolic disease remains lacking. In particular, it remains unclear whether EO and MS share a common intracellular lipid signature or exhibit distinct patterns that reflect disease progression.

MS is defined by a cluster of clinical and biochemical abnormalities, including central obesity, dyslipidemia, arterial hypertension, and insulin resistance [1,3]. These features are closely linked to chronic inflammation, often reflected by elevated levels of C-reactive protein (CRP) [17]. Understanding how PBMC sphingolipid metabolism integrates with these components may provide insight into the interplay between metabolic and immune pathways and help identify novel integrative biomarkers [18].

Therefore, the present study was designed with both exploratory and discriminative objectives. First, we performed an exploratory characterization of the PBMC sphingolipidomic profile in normal-weight healthy (NWH) subjects and in patients with EO or MS, using integrated univariate and multivariate approaches to identify patterns of lipid remodeling. Second, we explored associations between selected sphingolipid species and key cardiometabolic variables that align with the International Diabetes Federation (IDF) criteria for MS [19], including inflammatory status, to assess their potential biological relevance. Third, we evaluated the discriminative capacity of PBMC-derived sphingolipid signatures—both individual species and composite indices—using multivariate models to determine their ability to distinguish between metabolic phenotypes.

## 2. Materials and Methods

### 2.1. Subjects

Obese subjects (body mass index, BMI > 35 kg/m^2^), hospitalized at the Division of Metabolic Diseases, Istituto Auxologico Italiano, Piancavallo-Verbania, Italy, for a 3-week multidisciplinary integrated body weight reduction program (BWRP), were recruited for the current study. Normal-weight healthy (NWH) subjects, age-matched and selected among friends and relatives of the medical and nursing staff, were recruited as the control group. Both obese and NWH subjects were moderately active (60 min of physical activity, two times/week). All females were eumenorrheic; the study was conducted during the follicular phase of their menstrual cycle.

After verification of the exclusion criteria, particularly the presence of any disease other than essential obesity or the use of any drug, clinical, biochemical, and anthropometric data were collected from each participant, including evaluation of body composition by bioimpedance analysis (see below for details).

The study protocol was approved by the Ethical Committee (EC) of the Istituto Auxologico Italiano, IRCCS, Milan, Italy (EC code: 2021_02_23_11; research project: 01C126; acronym: SFINGOTRANSADIP). The protocol was explained to the subjects, who provided written informed consent. Participants were consecutively recruited between June 2021 and March 2022, based on the date of blood sample collection.

### 2.2. Clinical Assessment

#### 2.2.1. Anthropometric Measurements

A scale with a stadiometer was used to measure height and weight (Wunder Sa.Bi., WU150, Trezzo sull’Adda, Italy). Waist circumference (WC) was measured with a flexible tape in a standing position, halfway between the inferior margin of the ribs and the superior border of the crista. Body composition was measured by bioimpedance analysis (Human-IM Scan, DS-Medigroup, Milan, Italy) after 20 min of supine rest. BMI, fat mass (FM), and fat-free mass (FFM) were determined in all subjects.

#### 2.2.2. Blood Pressure

Blood pressure was measured on the right arm using a sphygmomanometer with an appropriate cuff size, with the subject seated and relaxed. The procedure was repeated three times at 10 min intervals; the means of the three values for systolic (SBP) and diastolic (DBP) blood pressure were recorded.

#### 2.2.3. Definition of Metabolic Syndrome and Essential Obesity

According to the IDF criteria for the diagnosis of metabolic syndrome in adults [19], MS was defined by the presence of central obesity plus at least two of the following four factors. Central obesity was defined as waist circumference (WC) ≥ 94 cm in males and ≥80 cm in females (European population-specific cut-offs). In addition, at least two of the following criteria were required: (i) hypertriglyceridemia: triglycerides (TG) ≥ 150 mg/dL (1.7 mmol/L), or specific treatment for this lipid abnormality; (ii) reduced HDL cholesterol (HDL-C): <40 mg/dL (1.03 mmol/L) in males and <50 mg/dL (1.29 mmol/L) in females, or specific treatment for this lipid abnormality; (iii) elevated blood pressure: systolic blood pressure (SBP) ≥ 130 mmHg and/or diastolic blood pressure (DBP) ≥ 85 mmHg, or treatment of previously diagnosed hypertension; (iv) impaired fasting glucose: fasting plasma glucose ≥ 100 mg/dL (5.6 mmol/L), or previously T2D. Obese subjects were classified as having MS when these criteria were met.

EO was defined as the presence of obesity in individuals who did not meet the minimum number of criteria required for the diagnosis of MS according to the IDF definition.

### 2.3. Laboratory Procedures

#### 2.3.1. Metabolic Variables

Fasting blood samples (~10 mL) were collected at approximately 8:00 AM after an overnight fast (~12 h) at the beginning of the BWRP.

Serum levels of total cholesterol (T-C), HDL-C, LDL cholesterol (LDL-C), TG, glucose, insulin, CRP, and glycated haemoglobin (HbA1c) were measured using standardized laboratory methods.

Lipid parameters (T-C, HDL-C, LDL-C, and TG) and glucose were measured using enzymatic colorimetric assays (Roche Diagnostics, Monza, Italy). Insulin was measured using a chemiluminescent immunometric assay (Elecsys Insulin, Roche Diagnostics), and CRP by immunoturbidimetric assay (<1 mg/L = low cardiovascular risk; 1–3 mg/L = moderate cardiovascular risk; >3 mg/L = high cardiovascular risk). HbA1c was quantified by high-performance liquid chromatography (HPLC) based on cation-exchange principles (Bio-Rad D-10 system), with calibration traceable to International Federation of Clinical Chemistry (IFCC) standards. Results were expressed as a percentage of total haemoglobin.

Assay sensitivities were within standard analytical ranges (≤9 mg/dL for lipids and triglycerides, 2 mg/dL for glucose, 0.2 µIU/mL for insulin, and 0.03 mg/dL for CRP). Analytical precision was confirmed by intra- and inter-assay coefficients of variation below 5% for all parameters.

Insulin resistance was estimated using the homeostatic model assessment (HOMA-IR), calculated as: insulin (µIU/mL) × glucose (mmol/L)/22.5 [20].

#### 2.3.2. PBMC Isolation from Whole Blood

PBMCs were isolated from fresh whole blood by density-gradient centrifugation using Ficoll-Paque, according to the manufacturer’s standard protocols. Whole blood collected in anticoagulant-treated tubes (heparin) was processed within 2 h after venipuncture to minimize cell degradation and ex vivo activation. Blood samples were diluted 1:1 with phosphate-buffered saline (PBS) or an equivalent balanced salt solution at room temperature, carefully layered over Ficoll-Paque PLUS (density 1.077 g/mL), and centrifuged at 400× *g* for 30 min at room temperature with the brake off. After centrifugation, the tubes exhibited different layers enriched in plasma, PBMC, Ficoll, and erythrocytes, respectively, from top to bottom. Once the plasma layer was removed, the PBMC layer at the plasma–Ficoll interface was carefully aspirated and transferred to a clean conical tube. Cells were washed twice with PBS by centrifugation at 300–400× *g* for 10 min to remove platelets and residual Ficoll. The final PBMC pellet was resuspended in PBS, counted, and immediately processed for sphingolipidomic analysis or stored at −80 °C until lipid extraction (see below). This procedure yields a PBMC-enriched fraction composed predominantly of lymphocytes and monocytes and is widely used for downstream immunological and omics applications.

#### 2.3.3. Lipid Extraction and Sphingolipid Content Quantification

Sphingolipid extraction and targeted LC–MS/MS analysis were performed as previously described [21,22]. Sphingolipids were assayed in 25 µL of PBMC suspension prepared as described above. This material was diluted to a final volume of 100 µL with water and, after the addition of 850 µL of a methanol/chloroform mixture (2:1 *v*/*v*), the samples were incubated for 1 h at 38 °C. Then, to enhance recovery, alkaline methanolysis was performed by incubating at 37 °C for 2 h with 75 µL of 1 M potassium hydroxide in methanol. After neutralization with 4 µL of pure acetic acid, samples were centrifuged (15 min at 13,400 rpm) and evaporated. The residues were dissolved in 100 µL of methanol, centrifuged for 10 min at 13,400 rpm, and transferred to a glass vial. Finally, samples were analyzed by LC Dionex 3000 UltiMate (Thermo Fisher Scientific, Waltham, MA, USA) coupled to a tandem mass spectrometer AB Sciex 3200 QTRAP (AB Sciex, Marlborough, MA, USA). The separation was achieved by reversed-phase chromatography, either using BEH C8 1.7 μm, 100 × 2.1 mm (for ceramides, dihydroceramides, and sphingomyelins) or Cortecs C18 1.6 μm, 100 × 2.1 mm (Waters, Milford, MA, USA) (for sphingoid bases) by mixing eluent A (0.2% formic acid, 2 mM ammonium formate water solution) and eluent B (methanol, 0.2% formic acid, 1 mM ammonium formate). Quantitative analysis was performed by interpolating each analyte peak area/internal standard area using a calibration curve for each sphingolipid.

#### 2.3.4. Quality Control and Analytical Validation of Lipidomic Measurements

To ensure the robustness, reproducibility, and analytical reliability of the sphingolipidomic data, a comprehensive set of quality control (QC) procedures was implemented throughout the experimental workflow, including sample preparation, LC–MS/MS acquisition, and data processing.

First, all samples were processed using a standardized and fully harmonized protocol for PBMC isolation and lipid extraction, minimizing pre-analytical variability. Sample handling times were tightly controlled, and all specimens were processed within 2 h of blood collection to reduce degradation and ex vivo metabolic alterations.

Quantitative lipidomic analysis was performed using internal standard–based calibration, as described above (Section 2.3.3). For each sphingolipid class, appropriate internal standards were added prior to extraction to correct for extraction efficiency, ionization variability, and instrument response. Calibration curves were generated for each analyte, and only signals within the linear dynamic range were considered for quantification.

To monitor analytical performance, pooled quality control samples were prepared by combining aliquots from multiple PBMC extracts and were periodically injected throughout the LC–MS/MS sequence. These QC samples were used to assess instrument stability, retention time consistency, and signal reproducibility across runs. The coefficient of variation (CV) for the main quantified sphingolipid species in QC samples was monitored and confirmed to be within acceptable analytical ranges.

Batch effects were minimized by analyzing samples in a randomized order across experimental runs. In addition, instrument performance was regularly checked using standard tuning procedures and evaluation of key parameters such as mass accuracy, sensitivity, and chromatographic resolution.

Although technical replicates at the extraction level were limited by sample availability, the combination of internal standard normalization, QC sample monitoring, and controlled analytical conditions ensured the reliability and comparability of the lipidomic measurements across all study groups.

### 2.4. Data Analysis

#### 2.4.1. Statistical Analysis and Software

Continuous variables were tested for normality using the Shapiro–Wilk test. As most sphingolipid concentrations were not normally distributed, data are presented as median and interquartile range (IQR), and non-parametric methods were used for group comparisons. Differences among groups (NWH subjects, and patients with EO or MS) were assessed using the Kruskal–Wallis test, followed by Dunn’s post hoc test for pairwise comparisons. When appropriate, to account for multiple testing across sphingolipid species, *p*-values were adjusted using the Benjamini–Hochberg false discovery rate (FDR) procedure.

Categorical variables are presented as counts or percentages. Differences across groups were assessed using the chi-square test.

Principal component analysis (PCA) was performed on the complete set of sphingolipid variables to explore the dataset’s global structure and to visualize sample clustering. Prior to PCA, data were log-transformed to reduce skewness and autoscaled (mean-centered and divided by the standard deviation) to ensure comparability across variables with different dynamic ranges. The proportion of explained variance by each principal component was reported.

Hierarchical clustering analysis was conducted using Euclidean distance and Ward’s linkage method on the subset of sphingolipids that were statistically significant after FDR correction in univariate group comparisons. Data were log-transformed and standardized prior to clustering. Heatmaps were generated to visualize patterns of relative abundance across samples, with rows representing subjects and columns representing lipid species.

Partial least squares discriminant analysis (PLS-DA) was performed using the subset of sphingolipids identified as significant in univariate analyses to evaluate multivariate discrimination among groups. Model performance was assessed using cross-validation, and variable importance in projection (VIP) scores were calculated to identify the most influential variables contributing to group separation.

Univariate linear regression analyses were conducted to investigate the association between sphingolipid species and cardiometabolic parameters, including BMI, WC, SBP and DBP, insulin resistance (HOMA-IR), HDL-C, TG, and CRP. Each sphingolipid was modeled as a dependent variable in separate ordinary least squares (OLS) regression models. Regression coefficients (β), standard errors (SE), 95% confidence intervals (CI), standardized coefficients (Std. β), and adjusted R^2^ values were reported. To account for multiple testing in regression analyses, *p*-values were adjusted using the Benjamini–Hochberg procedure applied separately within each predictor (i.e., across all sphingolipids tested for a given clinical variable), thereby controlling for the number of comparisons within each family of tests. This approach was chosen to balance type I error control with the correlation structure of lipidomic and clinical variables.

A two-sided *p*-value < 0.05 or FDR-adjusted *p*-value < 0.05, when appropriate, was considered statistically significant.

All statistical analyses were performed using Python (v. 3.14.4; Python Software Foundation, Wilmington, DE, USA). Data handling and preprocessing were conducted using Pandas (v. 3.0.2) and NumPy (v. 2.4.4). Group comparisons were performed using the SciPy library (v. 1.17.1). Multivariable linear regression models were computed using Statsmodels (v. 0.15.0). PCA was performed using Scikit-learn (v. 1.8.0). Hierarchical clustering and PLS-DA/VIP were generated using SciPy (v. 1.17.1) and Matplotlib (v. 3.10.9). Volcano plots, heatmaps, and boxplots were generated using Matplotlib (v. 3.10.9). All software listed above is available as open-source community software.

#### 2.4.2. Rationale for Variable Selection in Multivariate Analyses

In the present study, both exploratory and targeted statistical strategies were applied to the sphingolipidomic dataset in order to balance comprehensive pattern discovery with model interpretability and robustness.

For unsupervised exploratory analyses, including PCA and volcano plot visualization, the full set of quantified sphingolipid variables was retained (*n* = 38 individual species plus 3 ratio indices; total *n* = 41 variables). This choice was driven by the hypothesis-free nature of these approaches, which aim to capture the global structure of the lipidomic dataset without prior assumptions or variable selection. By including all measured sphingolipid variables, these analyses preserve the intrinsic covariance structure of the data and allow identification of dominant axes of variation reflecting coordinated metabolic remodeling.

In contrast, for supervised or structure-enhancing analyses, such as hierarchical clustering and PLS-DA, the variable space was restricted to the subset of sphingolipid variables that were statistically significant in univariate group comparisons after FDR correction (*n* = 10 variables). This selection was performed to reduce dimensionality relative to sample size, improve model stability, and limit the inclusion of noisy or non-informative variables that could obscure biologically meaningful patterns. Given the study’s moderate sample size, this approach was considered appropriate to mitigate overfitting and enhance the interpretability of clustering and discrimination results.

Similarly, univariate regression analyses of cardiometabolic variables were conducted using only the subset of previously significant sphingolipids (*n* = 10 variables). This targeted approach was adopted to focus on lipid species most likely to be biologically relevant and to reduce the multiple testing burden inherent in high-dimensional datasets. To further control the type I error rate, FDR correction was applied within each family of tests, as detailed above (Section 2.4.1).

#### 2.4.3. Missing Data

##### Extent of Missing Data

The proportion of missing data was generally low for clinical and anthropometric variables (<5%), while slightly higher levels were observed for selected biochemical and sphingolipid measurements (approximately 5–15%), likely reflecting technical and analytical variability. No variable showed excessive missingness.

##### Missing Data Handling Strategy

Missing values were handled using median imputation, given the relatively low proportion of missing data and the non-normal distribution of several variables. Median imputation was preferred over mean imputation to reduce the influence of skewed distributions and outliers.

This approach preserved the full sample size and avoided potential bias associated with a complete-case analysis.

##### Assumptions on Missingness

Based on the distribution of missing values across groups and variables, data were assumed to be missing at random (MAR).

##### Sensitivity Considerations

Given the limited proportion of missing data, median imputation is unlikely to have materially affected the results.

## 3. Results

### 3.1. Comparisons in Demographic, Clinical, and Biochemical Parameters

Table 1 summarizes the demographic, biochemical, and clinical characteristics of the NWH, EO, and MS groups, along with comparisons.

The three study groups were comparable in age and sex distribution, indicating appropriate matching and minimizing potential confounding effects of these variables.

As expected, the main differences were observed in anthropometric and metabolic parameters, reflecting a clear gradient of metabolic impairment. Both EO and MS groups showed markedly increased body weight, BMI, and FM compared with NWH subjects, confirming the presence of severe adiposity. Notably, WC further distinguished MS from EO, highlighting a more pronounced central fat distribution in the MS phenotype, consistent with its higher cardiometabolic risk profile.

Cardiovascular parameters followed a similar progressive pattern. HR and BP were already elevated in EO compared with NWH and further increased in MS, suggesting a stepwise worsening of hemodynamic regulation along the metabolic continuum.

Biochemical variables reinforced this gradient. HDL-C levels progressively declined from NWH to EO and MS, whereas TG and insulin levels, and HOMA-IR increased across groups, indicating worsening insulin sensitivity and lipid metabolism. Importantly, MS patients exhibited the most pronounced alterations, consistent with a more advanced metabolic derangement. HbA1c showed only modest differences, becoming significantly higher in MS, suggesting early glycemic impairment rather than overt dysglycemia in EO.

Inflammatory status, as reflected by CRP, was significantly elevated in both EO and MS compared with NWH, with no difference between the two conditions. This finding suggests that low-grade inflammation is already established in obesity and does not further increase substantially with the transition to MS.

In the Supplementary Material, Table S1 reports data on the prevalence of diabetes, hypertension, and dyslipidemia, as well as medication use, across NWH, EO, and MS groups.

### 3.2. Comparisons in Sphingolipid Species and Ratios

As shown in Table 2, among ceramides, Cer 18 and Cer 24:1 displayed significant differences after FDR correction. Both species exhibited a progressive increase from NWH to EO and MS, with the highest levels consistently observed in the MS group. Pairwise comparisons indicated that these differences were primarily driven by contrasts between NWH and MS, while EO values often occupied an intermediate position.

Other ceramide species, including Cer 16 and Cer 20, showed nominal significance at the unadjusted level but did not retain significance after FDR correction, suggesting a more modest or variable contribution.

Dihydroceramides showed a similar pattern of selective alteration. In particular, DHCer 16 and DHCer 18 were significantly elevated across groups, with increasing concentrations from NWH to EO and MS.

Other dihydroceramide species did not exhibit statistically robust differences after correction for multiple testing.

No sphingomyelin species remained significant after FDR adjustment, despite nominal differences observed for SM 18:1. This suggests that sphingomyelin metabolism is relatively preserved across the metabolic conditions studied, or that variability within groups masks subtle differences.

Among glycosphingolipids, HexCer 24:1, LacCer 16, and LacCer 24:1 emerged as significantly altered species. These lipids showed marked increases in EO and MS compared to NWH, with particularly pronounced elevations in MS.

Other HexCer and LacCer species showed either marginal or no significant differences after correction, reinforcing the specificity of the observed pattern.

Gangliosides (GM3 species) and sphingoid bases (Sph, S1P, DhSph, DhS1P) did not show significant differences after FDR correction.

Indices reflecting ceramide chain-length composition—CER16/24, CER18/24, and CER24:1/24—were among the most strongly associated variables, all remaining significant after FDR correction.

These ratios were markedly increased in EO and MS compared to NWH, indicating a shift in ceramide composition toward shorter or unsaturated species relative to very-long-chain ceramides. Importantly, these metrics exhibited some of the largest effect sizes, underscoring their potential as sensitive indicators of sphingolipid remodeling.

**Figure 1:**
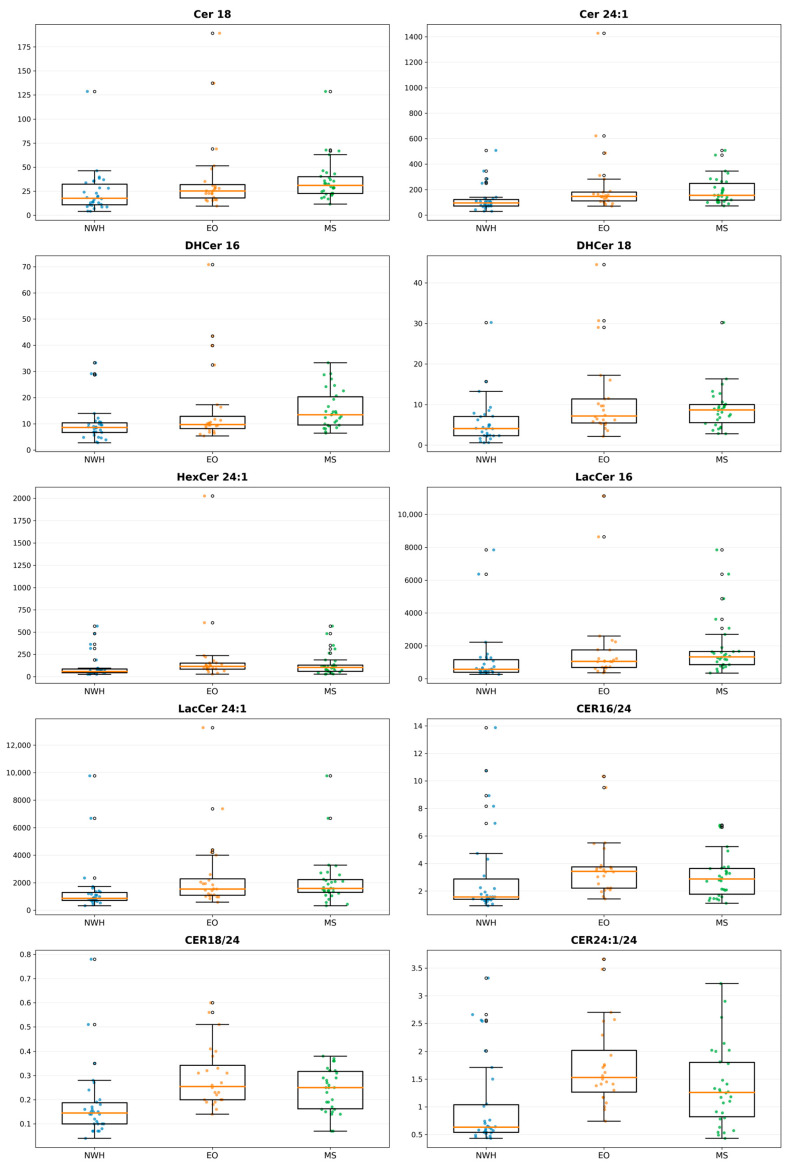
Boxplots of significant sphingolipid species and ratios across NWH, EO, and MS groups. Each panel represents one lipid variable. Boxes indicate the median and interquartile range, with whiskers representing distribution and dots individual values.

The boxplots (Figure 1) illustrate the heterogeneous distribution of individual significant sphingolipid variables across the three groups described above. Most of the significant species showed upward shifts in EO and/or MS relative to NWH, while the ratio variables (CER16/24, CER18/24, and CER24:1/24) also demonstrated clear group-level displacement. Taken together, the boxplots corroborated the univariate group comparisons and visually highlighted the broad lipidomic remodeling associated with EO and MS.

**Figure 2:**
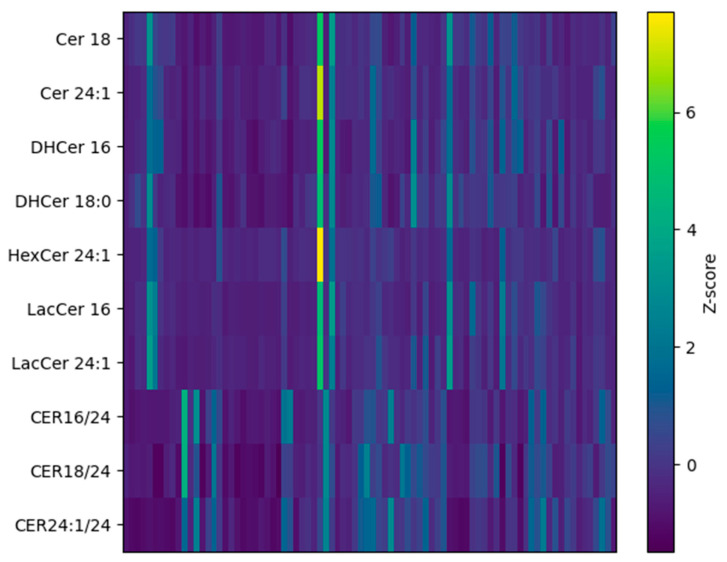
Heatmap of z-score standardized concentrations/values of significant sphingolipid species and ratios (after FDR correction) across NWH, EO, and MS. Rows represent lipids and columns represent subjects.

Further visualization is given by Figure 2, which shows the heatmap of z-scored concentrations/values for significant sphingolipid species/ratios. Overall, this visualization of sphingolipidomic results indicates progressive alterations in sphingolipid metabolism across groups, with EO representing an intermediate phenotype between NWH and MS. While several alterations are already evident in EO, the MS group exhibited a broader dysregulation of sphingolipid metabolism in PBMCs.

### 3.3. Univariate Regression Analyses

Univariate linear regression analyses restricted to sphingolipid species previously identified as significantly altered across study groups revealed a limited but coherent set of associations with specific cardiometabolic parameters (Table 3).

In particular, CER24:1/24 showed significant positive associations with both BMI and WC. Furthermore, DBP emerged as the only hemodynamic parameter significantly associated with multiple sphingolipid species. In fact, positive associations were observed for Cer 18; Cer 24:1, and DHCer 18.

Across all significant models, standardized regression coefficients were of moderate magnitude (Std. β ≈ 0.27–0.32), and adjusted R^2^ values ranged from approximately 0.06 to 0.09, indicating that individual predictors explained a modest proportion of variance in sphingolipid levels. Notably, all observed associations were positive, reflecting a consistent directional relationship between worsening cardiometabolic status (increased adiposity or elevated DBP) and higher sphingolipid concentrations or altered ceramide composition.

Table S2 (in the Supplementary Material) reports the full list of associations, including non-significant ones.

### 3.4. Exploratory Lipidomic Visualization: All Sphingolipid Variables

**Figure 3:**
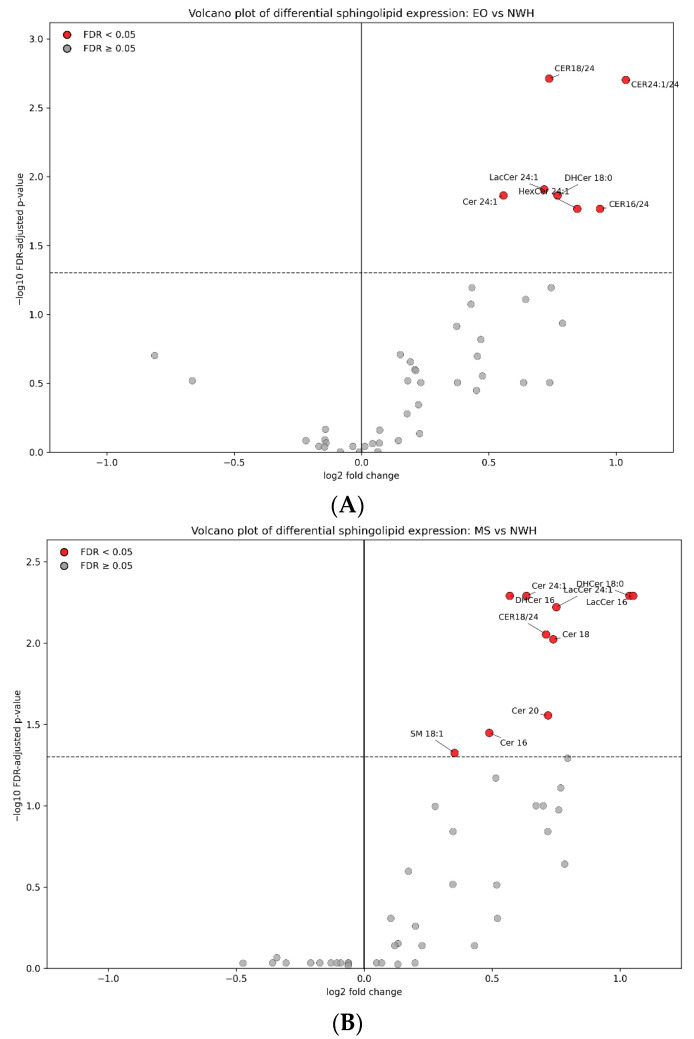
(**A**) Volcano plot showing differential leukocyte sphingolipid expression between EO and NWH. The x-axis represents the median-based log_2_ fold change (EO/NWH), and the y-axis represents −log_10_ of the Benjamini–Hochberg FDR-adjusted *p*-value. Significant lipids (FDR < 0.05) are highlighted and labeled. (**B**) Volcano plot showing differential leukocyte sphingolipid expression between MS and NWH. The x-axis represents the median-based log2 fold change (MS/NWH), and the y-axis represents −log10 of the Benjamini–Hochberg FDR-adjusted *p*-value. Significant lipids (FDR < 0.05) are highlighted and labeled. Dashed horizontal line indicates the FDR significance threshold (FDR = 0.05).

When comparing EO vs. NWH, the volcano plot identified 7 sphingolipid variables that remained significant after FDR correction. All significant features were shifted upward in EO relative to NWH, and no lipid met the significance threshold with a negative log_2_ fold change. The strongest EO-associated signals were the ceramide ratio indices CER18/24 (log_2_FC 0.737, FDR 0.0019) and CER24:1/24 (log_2_FC 1.038, FDR 0.0020). Additional significant elevations were observed for LacCer 24:1, DHCer 18:0, Cer 24:1, HexCer 24:1, and CER16/24. Taken together, the EO comparison was characterized mainly by increased very-long-chain ceramide remodeling and higher glycosphingolipid-related species or ratios rather than by a diffuse rise across the entire sphingolipidome (Figure 3A).

When comparing MS vs. NWH, the contrast yielded a broader differential signature, with 10 sphingolipid variables remaining significant after FDR correction. Again, all significant lipids showed positive log_2_ fold changes, indicating higher abundance in MS than in NWH. The most prominent signals were LacCer 16 (log_2_FC 1.050, FDR 0.0051) and DHCer 18:0 (log_2_FC 1.037, FDR 0.0051), closely accompanied by Cer 24:1, DHCer 16, LacCer 24:1, and CER18/24. In contrast to EO, the MS profile extended further to Cer 18, Cer 20, Cer 16, and SM 18:1, consistent with a more extensive sphingolipid perturbation associated with the metabolically more severe phenotype (Figure 3B).

**Figure 4:**
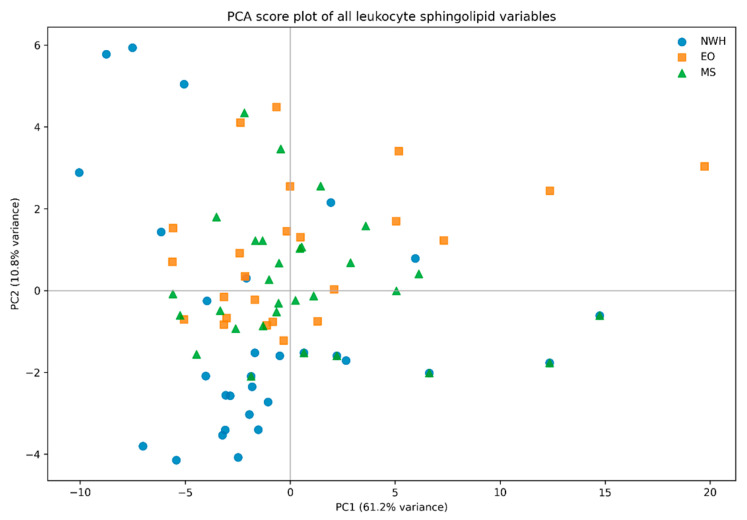
PCA score plot of all leukocyte sphingolipid variables after log_1_0 transformation and autoscaling. Grey reference lines indicate the zero coordinates of PC1 and PC2, corresponding to the origin of the PCA score space and facilitating visualization of sample distribution across quadrants.

When performing PCA (Figure 4), overall, PC1 explained 61.2% of the total variance, and PC2 explained 10.8%, yielding a cumulative two-component variance of 72.0%. Thus, the dominant structure of the full sphingolipid dataset was captured mainly by a strong first component and a more modest but still interpretable second component.

In terms of score-space separation, the corrected score plot showed that the strongest visible displacement occurred along PC1, where many EO and MS subjects shifted toward higher values relative to NWH, although overlap among the three groups remained substantial. Consistent with this visual pattern, the overall group comparison for PC1 did not reach conventional significance (Kruskal–Wallis *p* = 0.0875). By contrast, PC2 separated NWH from the two metabolically altered groups more clearly. The overall comparison for PC2 was significant (Kruskal–Wallis *p* = 0.0004808), indicating that the second component captured the most robust between-group structure in the full unsupervised model.

When focusing on pairwise exploratory comparisons, for PC1, no pairwise contrast remained significant after Holm adjustment: NWH vs. EO (Holm-adjusted *p* = 0.2921); NWH vs. MS (Holm-adjusted *p* = 0.09977); EO vs. MS (Holm-adjusted *p* = 0.5716). For PC2, significant differences were observed for NWH vs. EO and NWH vs. MS, whereas EO vs. MS was not significant after adjustment (Holm-adjusted *p* = 0.1036).

In terms of loading structure, PC1 behaved as a broad abundance-related sphingolipid axis. The largest absolute PC1 loadings were observed for GM 24:1, Cer 16, Cer 20, Cer 22, GM3 16, SM 16, GM3 18, and Cer 24:1, indicating that this component summarized coordinated variation across multiple ceramide, sphingomyelin, and ganglioside-related species rather than a narrow pathway-restricted signal. PC2 was driven positively mainly by CER16/24, CER24:1/24, CER18/24, SM 18:1, and HexCer 18:1, while the strongest negative contributors were DHCer 24, Cer 24, GM3 24, Cer 22, and GM3 22. This pattern indicates that PC2 contrasted ceramide-ratio remodeling against a set of longer-chain ceramide and glycosphingolipid features.

### 3.5. Clustering and Discrimination: Selected Sphingolipid Variables

**Figure 5:**
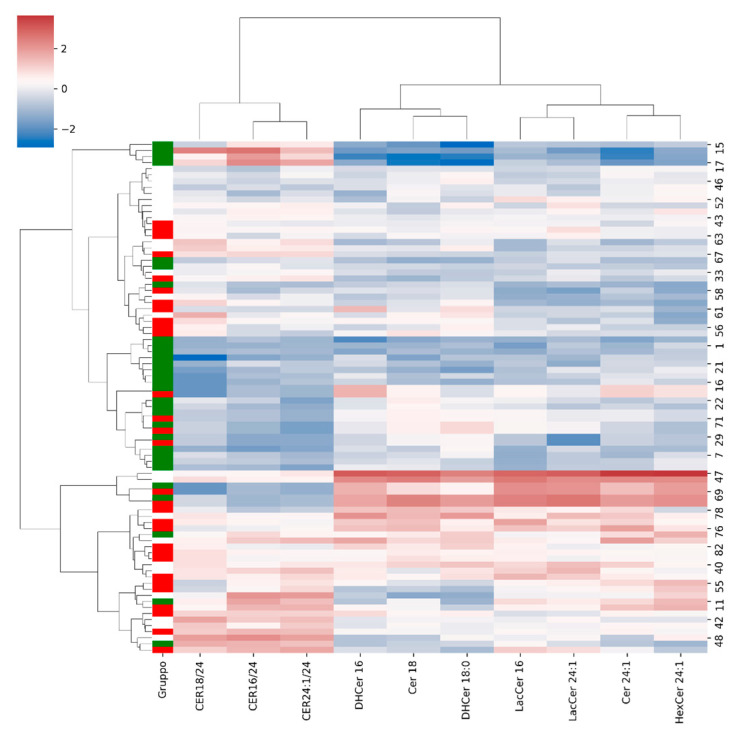
Hierarchical clustering heatmap of significant sphingolipid variables. Clustering reveals partial separation between NWH, EO, and MS, indicating overlapping lipidomic profiles.

Hierarchical clustering of the standardized significant sphingolipid matrix (Figure 5) revealed a clear separation between NWH subjects and EO/MS patients. EO and MS groups displayed partial overlap, supporting a metabolic continuum. The clustering pattern was driven by coordinated elevations in ceramides and glycosphingolipids, including Cer 18, Cer 24:1, DHCer 16, DHCer 18:0, HexCer 24:1, LacCer 16, and LacCer 24:1. Additionally, ceramide ratio indices (CER16/24, CER18/24, CER24:1/24) contributed significantly to the observed separation, reflecting alterations in sphingolipid chain-length distribution.

**Figure 6:**
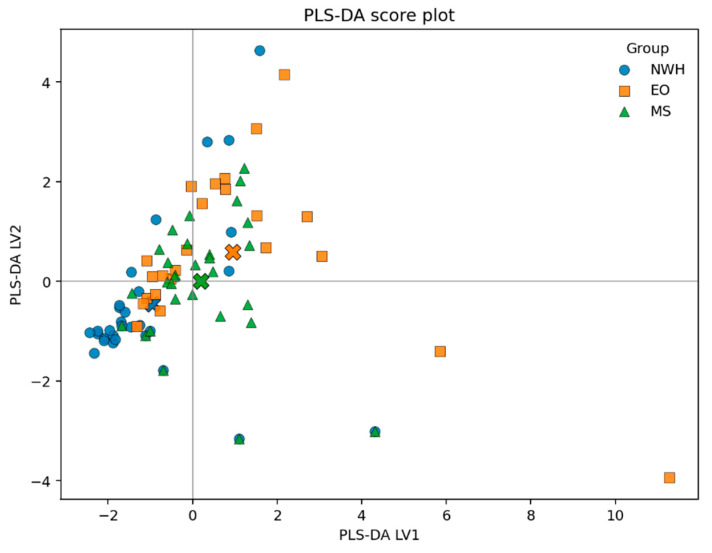
PLS-DA score plot of selected sphingolipid variables. Grey reference lines indicate the zero coordinates of the latent variables/components, facilitating visualization of sample distribution and group separation within the multivariate score space.

To further explore the multivariate structure of the data and assess whether the sphingolipid profile could discriminate among study groups, a PLS-DA was performed (Figure 6). The analysis was restricted to the subset of sphingolipid variables that remained statistically significant after FDR correction in the univariate analysis (*n* = 10), including both individual sphingolipid species and ratios.

Prior to modeling, all variables were mean-centered and scaled to unit variance to account for differences in magnitude and to ensure equal contribution to the model. Group membership (NWH-EO-MS) was encoded as a categorical response variable. The optimal number of latent components was determined through cross-validation, selecting the model that minimized classification error while avoiding overfitting.

The final PLS-DA model retained three latent components and showed a moderate ability to discriminate among the three groups. The overall classification accuracy was 0.548, with a balanced accuracy of 0.542, indicating performance above chance level but not strong separation. This was further supported by permutation testing (1000 permutations), which confirmed that the observed model performance was unlikely to have occurred by chance (*p* = 0.005).

Inspection of the cross-validated confusion matrix revealed that NWH subjects were classified with relatively high accuracy (26 out of 30 correctly assigned), whereas classification performance was lower for the EO and MS groups. In particular, a substantial degree of misclassification was observed between EO and MS, suggesting a partial overlap in their sphingolipid profiles. In contrast, NWH subjects appeared more clearly separated from the two pathological groups, indicating a more distinct metabolic phenotype.

**Figure 7:**
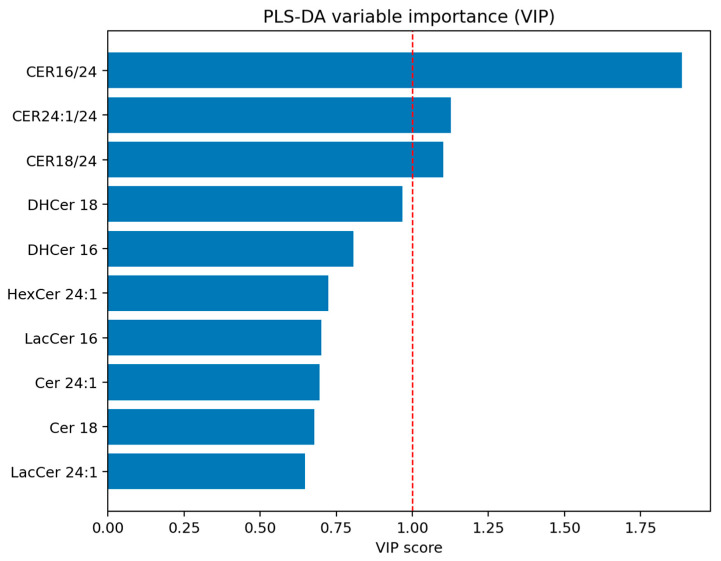
Variable importance in projection (VIP) scores from PLS-DA. Dashed red line indicates the threshold used to identify variables with the greatest contribution to group discrimination in the PLS-DA model (VIP score cutoff).

Evaluation of variable importance using VIP scores (Figure 7) indicated that ceramide ratios were the strongest contributors to group discrimination. In particular, CER16/24 showed the highest importance (VIP = 1.89), followed by CER24:1/24 and CER18/24 (both VIP > 1). These findings suggest that relative changes between ceramide species, rather than absolute concentrations alone, may better capture the metabolic alterations associated with EO and MS.

## 4. Discussion

### 4.1. PBMC Sphingolipidomics and Immunometabolic Remodeling

The present study demonstrates that sphingolipid profiling in PBMCs identifies a distinct intracellular immunometabolic signature associated with EO and MS [23].

Unlike circulating lipid measurements, which reflect systemic lipid transport and inter-organ exchanges, PBMC sphingolipidomics provides a direct insight into intracellular lipid metabolism within immune cells, thereby capturing processes more closely related to inflammation and metabolic reprogramming [24].

In this context, the observed alterations do not affect the entire sphingolipidome but are restricted to a specific subset of lipid species, indicating that metabolic dysfunction is characterized by selective and coordinated remodeling rather than a generalized increase in lipid abundance [25].

### 4.2. Ceramides, Dihydroceramides, and De Novo Synthesis Pathways

Among the altered lipid classes, ceramides and dihydroceramides emerged as central components of the metabolic signature [26].

The increase in Cer 18 and Cer 24:1, together with the elevation of DHCer 16 and DHCer 18, is consistent with activation of the de novo sphingolipid synthesis pathway, which is known to be upregulated in conditions of nutrient excess and lipotoxic stress [27].

Dihydroceramides, as intermediates in this pathway, are increasingly recognized as early indicators of metabolic perturbation, and their accumulation in PBMCs suggests that immune cells undergo metabolic reprogramming in response to systemic metabolic imbalance [28].

This pattern supports the hypothesis that intracellular ceramide metabolism in immune cells may actively contribute to the propagation of inflammatory responses associated with EO and MS [29].

### 4.3. Glycosphingolipids and Immune Cell Activation

In parallel with the increase in ceramides, significant elevations in glycosphingolipids, including HexCer 24:1, LacCer 16, and LacCer 24:1, indicate activation of downstream sphingolipid metabolic pathways [30].

These molecules play a critical role in membrane organization and signal transduction, particularly in immune cells [31].

Lactosylceramides, in particular, have been implicated in the activation of pro-inflammatory signaling cascades and oxidative stress pathways [32].

Their increased levels in PBMCs therefore suggest that sphingolipid remodeling is not only a metabolic phenomenon but is also functionally linked to immune activation, potentially contributing to the chronic low-grade inflammation that characterizes both EO and MS [33].

### 4.4. Ceramide Ratio Indices as Integrative Markers

A particularly relevant finding of the present study is the consistent alteration of ceramide ratio indices, including CER16/24, CER18/24, and CER24:1/24 [34].

These indices reflect changes in the relative composition of ceramide species rather than their absolute concentrations, providing insight into qualitative aspects of sphingolipid remodeling [35].

The observed increase in these ratios indicates a shift toward shorter-chain or monounsaturated ceramides relative to very long-chain saturated species, a pattern that may influence membrane properties and intracellular signaling pathways [36].

Notably, these ratio-based variables contributed strongly to multivariate models, highlighting their potential as sensitive integrative markers of metabolic dysfunction [37].

### 4.5. Metabolic Continuum from EO to MS

Multivariate analyses, including PCA and clustering approaches, revealed a clear separation between NWH subjects and patients with EO or MS, while also demonstrating a substantial overlap between EO and MS [38].

This pattern suggests that these conditions do not represent discrete categories but rather different stages along a continuum of metabolic dysregulation [39].

The progressive increase in sphingolipid alterations from NWH subjects to EO and MS further supports this interpretation, indicating that intracellular lipid remodelling intensifies as metabolic status worsens [40].

### 4.6. Limited Univariate Associations and Multivariate Complexity

Despite the clear differences observed at the group level, univariate regression analyses revealed only a limited number of significant associations between individual sphingolipids and cardiometabolic parameters after appropriate correction for multiple testing [41].

In particular, CER24:1/24 was associated with measures of adiposity, such as BMI and WC, whereas DBP showed associations with Cer 18, Cer 24:1, and DHCer 18 [42].

Although these associations were statistically significant and biologically plausible, their effect sizes were modest and explained only a limited proportion of the variance [43].

This apparent discrepancy between big group-level differences and weak univariate associations suggests that sphingolipid remodeling in PBMCs is not driven by single clinical variables but rather reflects the integrated influence of multiple interacting factors, with the limitation that, in the present study, only a limited number of (essentially cardiometabolic) factors were considered [44].

### 4.7. Dominance of Multivariate Lipid Signatures

Consistent with this interpretation, multivariate analyses demonstrated that the discrimination between study groups is driven by combined lipid signatures rather than by individual lipid species [45].

PCA indicated that overall lipid abundance contributes to the main axis of variation, while more subtle changes in ceramide composition are responsible for group separation [46].

Similarly, PLS-DA analyses showed only moderate classification performance, with partial overlap between EO and MS, further supporting the concept of shared metabolic pathways underlying these conditions [47].

These findings emphasize that sphingolipid metabolism operates as a coordinated network and that its dysregulation can be more effectively captured through multivariate approaches [48].

### 4.8. Biological and Clinical Implications

From a biological perspective, the observed sphingolipid profile likely reflects the combined effects of increased fatty acid availability, activation of de novo synthesis pathways, alterations in acyl-chain composition, and changes in membrane organization within immune cells [49].

These processes are closely linked to inflammatory signaling, suggesting that sphingolipid metabolism may represent a key interface between metabolic stress and immune activation [50].

From a clinical standpoint, the identification of a selective intracellular sphingolipid signature suggests that PBMC lipid profiling may provide novel biomarkers of metabolic dysfunction [51].

Importantly, the results indicate that composite lipid signatures or ratio-based indices may be more informative than individual lipid species, with potential implications for risk stratification and therapeutic targeting [52,53,54].

### 4.9. Limitations and Future Directions

Several limitations should be considered. The cross-sectional design precludes causal inference, and although the sample size is adequate for exploratory lipidomic analyses, it may limit the detection of weaker associations.

A further limitation of this study is the selection of NWH controls among friends and relatives of the medical and nursing staff, which may introduce selection bias and limit the generalizability of the findings to the broader population.

In addition, PBMCs represent a heterogeneous population of immune cells, and cell-specific lipid alterations could not be resolved. Future studies incorporating cell-type–specific analyses and longitudinal designs will be necessary to further elucidate the role of sphingolipid metabolism in immunometabolic regulation.

A final limitation of the present study relates to the interpretative framework adopted in the Discussion. Although we attempted to contextualize our findings within a plausible pathophysiological framework linking PBMC sphingolipid remodeling to immunometabolic dysfunction, some of these interpretations remain inherently tentative and partly mechanistic. Given the cross-sectional design of the study, no temporal or causal relationships can be established, and the observed associations should not be interpreted as evidence of underlying biological mechanisms. Rather, these interpretations should be considered hypothesis-generating and require confirmation in future longitudinal or prospective studies, which are better suited to defining cause–and–effect relationships and validating the proposed mechanistic links.

## 5. Conclusions

The present study demonstrates that sphingolipidomic profiling of PBMCs identifies a selective and coordinated intracellular lipid signature associated with EO and MS. Unlike conventional plasma-based analyses, this approach captures immune cell-specific metabolic remodeling, providing a complementary and potentially more informative perspective on the interplay between metabolism and inflammation.

A key finding is that sphingolipid alterations are not generalized but rather involve a restricted subset of ceramides, dihydroceramides, glycosphingolipids, and ceramide ratio indices, indicating a structured reprogramming of intracellular lipid metabolism. In particular, ceramide ratios emerged as strong contributors to group discrimination, supporting their role as integrative indicators of sphingolipid remodeling. Multivariate analyses further indicate that EO and MS share substantial overlap in their lipidomic profiles, consistent with a metabolic continuum, while remaining distinguishable from NWH subjects.

Importantly, the study highlights two conceptually distinct but complementary implications. From a biomarker perspective, PBMC sphingolipid signatures—especially ratio-based indices and multivariate patterns—may serve as integrative markers of metabolic dysfunction, reflecting the combined influence of adiposity, inflammation, and cardiometabolic alterations. From a mechanistic perspective, the observed lipid changes provide evidence of intracellular immunometabolic reprogramming, supporting the role of sphingolipid metabolism as a link between metabolic stress and immune activation. However, given the cross-sectional and exploratory design, these mechanistic insights should be considered hypothesis-generating rather than definitive.

Notably, the limited associations observed in univariate regression analyses suggest that sphingolipid alterations are not driven by single clinical variables but instead reflect complex, multidimensional metabolic interactions, reinforcing the relevance of multivariate approaches in lipidomic research.

Overall, the main take-home message of this study is that PBMC-based sphingolipidomics represents a novel and informative framework for investigating immunometabolic dysfunction, revealing intracellular processes not detectable through circulating lipid measurements alone.

Future studies are warranted to validate these findings in independent and larger cohorts, to develop predictive models that integrate lipidomic and clinical data, and to explore the therapeutic potential of targeting sphingolipid pathways, particularly those related to ceramide metabolism and immune cell activation. Longitudinal and mechanistic investigations will be essential to clarify causal relationships and to establish the clinical utility of these biomarkers in metabolic disease.

## Figures and Tables

**Table 1:** Descriptive Statistics and Group Comparisons of demographic, biochemical and clinical parameters in normal-weight (NWH) subjects and patients with essential obesity (EO) or metabolic syndrome (MS).

Variable	NWH	EO	MS	*p*_Overall	NWH vs. EO	NWH vs. MS	EO vs. MS	Effect Size (ε^2^) [95% CI]
n.	30	24	30	/	/	/	/	/
Sex (n, %) Female Male	19 (63%) 11 (37%)	18 (75%) 6 (25%)	19 (63%) 11 (37%)	0.5916	/	/	/	/
Age (years)	29.15 (26.42–33.14)	27.38 (21.35–35.65)	30.43 (23.97–41.17)	0.5585	0.3253	0.9705	0.3699	0.000 [0.000–0.103]
Weight (kg)	68.00 (58.58–72.83)	118.45 (107.97–129.23)	124.75 (113.60–132.20)	0.0000	0.0000	0.0000	0.2007	0.690 [0.588–0.749]
Height (m)	1.71 (0.09)	1.66 (0.07)	1.68 (0.11)	0.2105	0.0495	0.3109	0.4864	0.015 [0.000–0.123]
BMI (kg/m^2^)	22.85 (20.77–24.70)	42.85 (40.77–45.70)	43.45 (41.55–46.58)	0.0000	0.0000	0.0000	0.4083	0.685 [0.587–0.751]
WC (cm)	78.00 (76.25–82.75)	110.00 (106.00–119.25)	120.00 (113.25–126.50)	0.0000	0.0000	0.0000	0.0036	0.725 [0.610–0.782]
FFM (kg)	53.05 (46.30–59.00)	55.65 (50.80–63.58)	61.85 (53.80–66.03)	0.0075	0.0947	0.0023	0.1611	0.096 [0.003–0.209]
FFM (%)	79.45 (73.97–82.30)	47.40 (44.88–53.08)	48.75 (43.67–52.73)	0.0000	0.0000	0.0000	0.9583	0.662 [0.558–0.741]
FM (kg)	13.15 (10.80–17.93)	60.25 (52.80–67.10)	61.00 (57.27–68.07)	0.0000	0.0000	0.0000	0.5598	0.631 [0.496–0.746]
FM (%)	20.20 (17.52–26.02)	52.60 (46.92–55.12)	51.25 (47.40–56.52)	0.0000	0.0000	0.0000	0.8686	0.662 [0.558–0.741]
SBP (mmHg)	120.00 (110.00–120.00)	120.00 (120.00–130.00)	130.00 (130.00–140.00)	0.0000	0.0015	0.0000	0.0008	0.429 [0.292–0.611]
DBP (mmHg)	70.00 (70.00–75.00)	80.00 (77.50–80.00)	80.00 (80.00–90.00)	0.0000	0.0003	0.0000	0.0137	0.434 [0.288–0.585]
HR (beats/min)	70.00 (69.00–72.00)	80.00 (77.50–90.00)	88.00 (84.25–96.75)	0.0000	0.0000	0.0000	0.0498	0.557 [0.468–0.658]
mREE (Kcal)	1572.50 (1378.75–1845.75)	1902.50 (1804.00–2245.75)	2070.50 (1852.25–2308.50)	0.0003	0.0019	0.0003	0.3211	0.175 [0.024–0.364]
Glucose (mg/dL)	87.00 (82.25–94.25)	83.00 (80.00–88.25)	86.00 (82.25–94.75)	0.2745	0.1186	0.5688	0.2836	0.007 [0.000–0.121]
Insulin (mU/L)	6.65 (5.12–8.80)	15.85 (11.00–23.55)	25.05 (19.07–30.25)	0.0000	0.0000	0.0000	0.0028	0.490 [0.275–0.657]
HOMA-IR	1.54 (1.06–1.83)	3.22 (2.23–4.68)	5.30 (4.32–6.21)	0.0000	0.0000	0.0000	0.0013	0.458 [0.237–0.618]
T-C (mg/dL)	179.87 (34.30)	161.79 (35.66)	169.77 (34.40)	0.1653	0.0642	0.2595	0.4087	0.014 [0.000–0.131]
HDL-C (mg/dL)	65.00 (56.25–70.75)	45.50 (39.50–50.25)	37.50 (32.50–43.75)	0.0000	0.0000	0.0000	0.0024	0.557 [0.413–0.727]
LDL-C (mg/dL)	108.50 (33.11)	100.04 (32.66)	111.30 (28.46)	0.4086	0.3524	0.7267	0.1820	0.000 [0.000–0.102]
TG (mg/dL)	63.00 (53.00–85.75)	96.00 (85.75–123.25)	125.50 (103.50–159.25)	0.0000	0.0004	0.0000	0.0128	0.369 [0.207–0.529]
HbA_1c_ (%)	5.10 (5.00–5.30)	5.10 (5.00–5.40)	5.40 (5.10–5.60)	0.0326	0.5982	0.0167	0.0488	0.060 [0.000–0.220]
CRP (mg/dL)	0.10 (0.00–0.20)	0.50 (0.28–1.03)	0.55 (0.40–1.08)	0.0000	0.0000	0.0000	0.4263	0.419 [0.289–0.585]

Note: Continuous variables were tested for normality using the Shapiro–Wilk test. As most sphingolipid concentrations were not normally distributed, data are presented as median and interquartile range (IQR), and non-parametric methods were applied for group comparisons. Differences among groups (NWH subjects, and patients with EO or MS) were assessed using the Kruskal–Wallis test, followed by Dunn’s post hoc test for pairwise comparisons. Categorical variables are presented as counts or percentages. Differences across groups were assessed using the chi-square test. For abbreviations, see the abbreviation list.

**Table 2:** Descriptive Statistics and Group Comparisons of leukocyte sphingolipids in normal-weight (NWH) subjects and patients with essential obesity (EO) or metabolic syndrome (MS).

Variable	NWH	EO	MS	*p*_Overall	*p*_Overall_FDR	NWH vs. EO	NWH vs. SM	EO vs. SM	Effect Size (ε^2^) [95% CI]
Cer 14	5.62 (4.12–6.88)	5.00 (4.16–6.64)	6.20 (4.26–9.29)	0.5968	0.8377	0.7428	0.7321	0.7321	0.000 [0.000–0.085]
Cer 16	238.53 (166.45–329.88)	279.22 (231.58–419.42)	362.12 (280.27–517.28)	0.0207	0.0772	0.2077	0.0161	0.2077	0.071 [0.000–0.199]
Cer 18:1	5.05 (3.22–8.00)	6.03 (3.91–9.84)	5.48 (4.12–8.69)	0.4258	0.6754	0.4257	0.4257	0.8776	0.000 [0.000–0.134]
Cer 18	17.80 (10.93–32.44)	25.42 (18.03–32.00)	31.08 (22.83–40.11)	0.0039	0.0226	0.0897	0.0028	0.2164	0.112 [0.017–0.283]
Cer 20	32.64 (20.71–58.89)	46.38 (30.99–57.73)	55.38 (41.81–73.37)	0.0175	0.0730	0.1892	0.0137	0.2521	0.075 [0.000–0.238]
Cer 22	109.62 (73.12–135.60)	93.37 (66.28–123.99)	101.35 (80.05–143.99)	0.6794	0.8666	0.7548	0.7548	0.7548	0.000 [0.000–0.061]
Cer 24:1	95.31 (71.71–123.05)	147.19 (111.38–180.89)	156.03 (117.57–249.88)	0.0004	0.0036	0.0029	0.0007	0.7203	0.171 [0.025–0.373]
Cer 24	148.46 (96.45–204.91)	91.63 (68.03–155.70)	119.05 (97.28–216.35)	0.2723	0.4682	0.2729	0.8655	0.2729	0.007 [0.000–0.131]
DHCer 16	8.65 (6.73–10.43)	9.77 (8.24–12.91)	13.51 (9.60–20.36)	0.0013	0.0085	0.1039	0.0008	0.1039	0.139 [0.038–0.267]
DHCer 18:1	0.62 (0.00–0.91)	1.10 (0.82–1.37)	1.12 (0.64–1.73)	0.0308	0.0886	0.0381	0.0381	0.8560	0.061 [0.000–0.219]
DHCer 18	4.09 (2.35–7.09)	7.18 (5.48–11.42)	8.70 (5.57–10.04)	0.0004	0.0036	0.0014	0.0014	0.9915	0.171 [0.053–0.333]
DHCer 24:1	27.80 (21.33–48.67)	36.89 (27.95–51.92)	41.22 (31.63–67.37)	0.0580	0.1482	0.2541	0.0536	0.3904	0.046 [0.000–0.188]
DHCer 24	59.59 (25.97–87.69)	33.44 (17.27–45.35)	42.59 (25.72–101.72)	0.1288	0.2913	0.1291	0.8905	0.1291	0.026 [0.000–0.189]
SM 16	8172.08 (6924.46–11,109.94)	8675.61 (6453.09–12,107.28)	9696.94 (6908.30–12,972.66)	0.6191	0.8377	0.6507	0.6507	0.6507	0.000 [0.000–0.080]
SM 18	1271.60 (981.65–1698.78)	1345.72 (1036.89–2107.25)	1594.06 (1363.28–1999.15)	0.1221	0.2913	0.4203	0.1232	0.3939	0.027 [0.000–0.164]
SM 18:1	497.63 (303.18–686.35)	672.20 (547.46–985.98)	665.35 (533.86–944.80)	0.0218	0.0772	0.0384	0.0380	0.9057	0.070 [0.000–0.233]
SM 24	3850.51 (2928.45–5246.75)	3444.31 (2376.08–4986.58)	3661.93 (2786.78–5107.30)	0.7768	0.9162	0.7629	0.7629	0.7629	0.000 [0.000–0.085]
SM 24:1	5625.44 (4029.50–7200.88)	6730.08 (5098.75–8910.05)	6530.73 (4968.32–9649.68)	0.1707	0.3570	0.1649	0.1649	0.9047	0.019 [0.000–0.151]
HexCer 16	28.01 (21.92–39.28)	51.95 (33.05–72.68)	50.78 (28.30–88.50)	0.0469	0.1268	0.0567	0.0567	0.9711	0.051 [0.000–0.181]
HexCer 18	2.72 (1.76–3.58)	3.83 (2.21–5.60)	4.10 (2.06–6.70)	0.2748	0.4682	0.3911	0.3686	0.7414	0.007 [0.000–0.113]
HexCer 18:1	3.27 (2.11–4.12)	3.39 (2.03–4.70)	2.77 (2.10–3.91)	0.8515	0.9793	0.8025	0.8025	0.8025	0.000 [0.000–0.056]
HexCer 20	4.55 (2.73–7.43)	5.42 (3.11–8.59)	6.76 (3.41–10.32)	0.4761	0.7064	0.5960	0.5960	0.5960	0.000 [0.000–0.099]
HexCer 22	36.73 (21.07–55.14)	32.49 (21.54–50.07)	34.34 (20.15–47.82)	0.9370	0.9821	0.9473	0.9473	0.9473	0.000 [0.000–0.087]
HexCer 24	46.46 (26.64–72.86)	41.86 (34.08–62.34)	36.33 (26.91–49.14)	0.7113	0.8666	0.8204	0.8204	0.8204	0.000 [0.000–0.072]
HexCer 24:1	58.69 (44.46–86.33)	115.75 (85.49–153.32)	103.30 (60.91–130.45)	0.0065	0.0331	0.0063	0.0475	0.2947	0.100 [0.000–0.269]
LacCer 16	575.02 (397.07–1162.74)	1052.08 (690.51–1751.78)	1331.83 (853.29–1662.09)	0.0010	0.0075	0.0264	0.0008	0.2873	0.146 [0.027–0.296]
LacCer 18	49.08 (36.31–81.72)	70.19 (56.15–120.91)	90.25 (51.08–118.56)	0.0250	0.0799	0.0545	0.0358	0.7818	0.066 [0.002–0.204]
LacCer 18:1	2.46 (1.90–4.82)	3.45 (2.42–5.83)	4.39 (2.21–5.66)	0.1330	0.2913	0.1303	0.1303	0.9379	0.025 [0.000–0.159]
LacCer 20	154.78 (95.66–247.79)	181.58 (151.09–279.55)	200.59 (141.11–290.70)	0.2040	0.4080	0.2070	0.2070	0.9990	0.015 [0.000–0.114]
LacCer 22	1082.02 (740.95–1451.74)	1054.66 (766.81–1350.77)	951.89 (750.71–1505.37)	0.9585	0.9821	0.8840	0.8840	0.8840	0.000 [0.000–0.046]
LacCer 24	711.73 (546.50–1052.93)	707.71 (469.88–1006.10)	680.62 (480.33–1018.35)	0.9043	0.9821	0.9851	0.9851	0.9851	0.000 [0.000–0.060]
LacCer 24:1	876.80 (726.19–1293.83)	1547.18 (1093.51–2287.87)	1587.40 (1312.08–2237.32)	0.0004	0.0036	0.0015	0.0014	0.9960	0.169 [0.047–0.312]
GM3 16	194.17 (137.60–225.36)	227.34 (183.75–344.16)	216.88 (155.17–303.05)	0.2413	0.4625	0.2765	0.3866	0.3866	0.010 [0.000–0.136]
GM3 18	17.70 (12.62–26.54)	21.34 (16.24–31.09)	21.21 (14.21–32.19)	0.4614	0.7064	0.5956	0.5956	0.6861	0.000 [0.000–0.112]
GM3 18:1	0.98 (0.14–1.58)	0.87 (0.62–1.65)	0.72 (0.51–1.67)	0.7159	0.8666	0.7947	0.7947	0.7947	0.000 [0.000–0.095]
GM3 20	35.02 (22.46–69.57)	40.24 (29.27–73.64)	48.77 (25.10–100.70)	0.6056	0.8377	0.5956	0.5956	0.9423	0.000 [0.000–0.140]
GM3 22	281.51 (227.26–419.20)	294.94 (203.42–452.38)	309.86 (214.57–414.47)	0.9945	0.9945	0.9831	0.9831	0.9831	0.000 [0.000–0.076]
GM3 24	66.67 (44.64–150.17)	74.12 (54.45–112.78)	60.61 (36.56–111.61)	0.6594	0.8666	0.7428	0.7428	0.7428	0.000 [0.000–0.089]
GM 24:1	243.34 (193.64–335.56)	342.02 (295.60–490.61)	320.05 (248.04–376.30)	0.0261	0.0799	0.0267	0.1018	0.3711	0.065 [0.000–0.239]
Sph	1093.64 (737.40–1365.50)	1103.86 (608.02–1700.84)	1151.88 (651.31–1595.33)	0.9607	0.9821	0.9672	0.9672	0.9672	0.000 [0.000–0.058]
S1P	81.35 (55.96–133.85)	137.67 (56.07–174.71)	84.41 (46.34–146.23)	0.3704	0.6085	0.4052	0.7995	0.4052	0.000 [0.000–0.118]
DhSph	167.36 (122.22–224.16)	157.49 (120.52–260.49)	194.30 (101.75–250.21)	0.9259	0.9821	0.9851	0.9851	0.9851	0.000 [0.000–0.079]
DhS1P	33.05 (21.18–46.99)	52.05 (23.78–62.90)	31.55 (15.68–51.75)	0.2581	0.4682	0.2380	0.9325	0.2380	0.009 [0.000–0.132]
CER16/24	1.57 (1.40–2.89)	3.43 (2.22–3.76)	2.88 (1.77–3.65)	0.0074	0.0342	0.0058	0.0976	0.1733	0.096 [0.000–0.308]
CER18/24	0.15 (0.10–0.19)	0.26 (0.20–0.34)	0.25 (0.16–0.32)	0.0001	0.0027	0.0001	0.0020	0.2445	0.216 [0.046–0.436]
CER24:1/24	0.64 (0.54–1.04)	1.53 (1.27–2.02)	1.26 (0.82–1.80)	0.0002	0.0036	0.0001	0.0253	0.0647	0.186 [0.041–0.397]

Note: Continuous variables were summarized as median (interquartile range, IQR). Differences among the three study groups were assessed using the Kruskal–Wallis rank-sum test, as suggested by the nonparametric distributional summary and the reporting of a global *p*-value (*p* overall) for three-group comparisons. To account for multiple testing across analytes, the resulting overall *p*-values were adjusted using the false discovery rate (FDR) approach according to the Benjamini–Hochberg procedure, and the adjusted values are reported as *p* overall FDR. When appropriate, post hoc pairwise comparisons between groups were performed using Dunn’s test for multiple nonparametric comparisons, yielding the pairwise *p*-values for NWH vs. EO, NWH vs. MS, and EO vs. MS. Effect size for the overall group comparison was estimated using epsilon-squared (ε^2^), with corresponding 95% confidence intervals. For abbreviations see the abbreviation list.

**Table 3:** Significant univariate regressions for selected lipids.

Sphingolipid Variable	Predictor	Beta	SE	95% CI	Std.Beta	*p*	AdjR2	*p*_FDR
CER24:1/24	BMI	0.0211	0.0069	0.007 to 0.035	0.3185	0.0031	0.0905	0.0315
CER24:1/24	WC	0.012	0.004	0.004 to 0.020	0.3134	0.0037	0.0872	0.037
Cer 18	DBP	1.003	0.33	0.346 to 1.659	0.3182	0.0032	0.0903	0.0219
Cer 24:1	DBP	5.2393	2.0345	1.192 to 9.287	0.2735	0.0118	0.0635	0.0394
DHCer 18	DBP	0.2432	0.083	0.078 to 0.408	0.3079	0.0044	0.0837	0.0219

Note: Univariate linear regression analyses were performed to assess the association between each sphingolipid variable and individual cardiometabolic predictors, including body mass index (BMI), waist circumference (WC), systolic and diastolic blood pressure (SBP, DBP), insulin resistance (HOMA-IR), lipid profile parameters (HDL-C and triglycerides), and C-reactive protein (CRP). Each sphingolipid was modeled as a dependent variable in separate ordinary least squares (OLS) regression models. Regression coefficients (β), standard errors (SE), 95% confidence intervals (CI), standardized coefficients (Std. β), and adjusted R^2^ values are reported. To account for multiple testing, *p*-values were adjusted using the Benjamini–Hochberg false discovery rate (FDR) procedure applied separately within each predictor (i.e., across all sphingolipids tested for a given predictor), thereby controlling for the number of comparisons within each family of tests. Only sphingolipid species previously identified as statistically significant in group-wise comparisons were included in this analysis. A two-sided FDR-adjusted *p*-value < 0.05 was considered statistically significant. See Table S2 for the full list of associations. For abbreviations, see the abbreviation list.

## Data Availability

The datasets used and/or analyzed in the present study are available from the corresponding author upon reasonable request. Raw data will be uploaded to Zenodo immediately after the manuscript is accepted.

## Supplementary Materials

The following supporting information can be downloaded at: https://www.mdpi.com/article/10.3390/jcm15103634/s1, Table S1: Distribution of Pharmacological Treatments and Metabolic Syndrome Criteria Across Study Groups; Table S2: All regressions (significant lipids, FDR per predictor).

## Abbreviations

ANOVA, analysis of variance; BMI, body mass index; BWRP, body weight reduction program; Cer, ceramides; CRP, C-reactive protein; CV, coefficient of variation; DBP, diastolic blood pressure; DHCer, dihydroceramides; EC, Ethical Committee; EO, essential obesity; FFM, fat-free mass; FM, fat mass; GM, gangliosides; HDL-C, high-density lipoprotein cholesterol; HexCer, hexosylceramides; HOMA-IR, homeostatic model assessment of insulin resistance; HPLC, high-performance liquid chromatography; HR, heart rate; IDF, International Diabetes Federation; IFCC, International Federation of Clinical Chemistry; LacCer, lactosylceramides; LC–MS/MS, liquid chromatography–tandem mass spectrometry; LDL-C, low-density lipoprotein cholesterol; MS, metabolic syndrome; NWH, normal-weight healthy; OLS, ordinary least squares; PBMC, peripheral blood mononuclear cells; PBS, phosphate-buffered saline; PCA, principal component analysis; PLS-DA, partial least squares discriminant analysis; QC, quality control; SBP, systolic blood pressure; SM, sphingomyelins; T2D, type 2 diabetes; T-C, total cholesterol; TG, triglycerides; VIP, variable importance in projection; WC, waist circumference.
