# Supplementary material for: Sphingolipidomic Profiling of Peripheral Blood Mononuclear Cells Reveals a Distinct Immunometabolic Signature Across Patients with Essential Obesity and Metabolic Syndrome Compared to Normal-Weight Healthy Subjects
Source: J Clin Med. 2026 May 9;15(10):3634. doi: 10.3390/jcm15103634 (PMC13207301; doi:10.3390/jcm15103634)
Supplement: Supplementary file 1 [file jcm-15-03634-s001.zip › Table S1.pdf]

Table S1. Distribution of Pharmacological Treatments and Metabolic Syndrome Criteria Across Study Groups.

Variable	NWH	NW	EO	EO	MS	MS
	Yes (n, %)	No (n, %)	Yes (n, %)	No (n, %)	Yes (n, %)	No (n, %)
Glucose-lowering drugs	0 (0.0%)	30 (100%)	0 (0.0%)	24 (100%)	2 (6.7%)	28 (93.3%)
Antihypertensive drugs	0 (0.0%)	30 (100%)	0 (0.0%)	24 (100%)	2 (6.7%)	28 (93.3%)
Lipid-lowering drugs	0 (0.0%)	30 (100%)	0 (0.0%)	24 (100%)	0 (0.0%)	30 (100%)
WC	0 (0.0%)	30 (100%)	24 (100%)	0 (0.0%)	30 (100%)	0 (0.0%)
TG	0 (0.0%)	30 (100%)	2 (8.3%)	22 (91.7%)	12 (40.0%)	18 (60.0%)
HDL-C	0 (0.0%)	30 (100%)	10 (41.7%)	14 (58.3%)	27 (90.0%)	3 (10.0%)
BP (SBP/DBP)	0 (0.0%)	30 (100%)	8 (33.3%)	16 (66.7%)	26 (86.7%)	4 (13.3%)
Glucose	0 (0.0%)	30 (100%)	0 (0.0%)	24 (100%)	9 (30.0%)	21 (70.0%)

Data are expressed as number (percentage). The cut-off values for waist circumference (WC), triglycerides (TG), HDL cholesterol (HDL-C), blood pressure (BP), and fasting glucose are defined according to the International Diabetes Federation (IDF) criteria for metabolic syndrome. See the text for further details.
