# Supplementary material for: Sphingolipidomic Profiling of Peripheral Blood Mononuclear Cells Reveals a Distinct Immunometabolic Signature Across Patients with Essential Obesity and Metabolic Syndrome Compared to Normal-Weight Healthy Subjects
Source: J Clin Med. 2026 May 9;15(10):3634. doi: 10.3390/jcm15103634 (PMC13207301; doi:10.3390/jcm15103634)
Supplement: Supplementary file 1 [file jcm-15-03634-s001.zip › Table S2.pdf]

Table S2. All regressions (significant lipids, FDR per predictor).

Lipid	Predictor	Beta	SE	95% CI	Std.Beta	p	AdjR2	p_FDR
Cer 18	BMI	0.4233	0.2632	-0.100 to 0.947	0.1749	0.1116	0.0188	0.1483
Cer 24:1	BMI	3.4511	1.5789	0.310 to 6.592	0.2346	0.0317	0.0435	0.1027
DHCer 16	BMI	0.1795	0.0938	-0.007 to 0.366	0.2067	0.0592	0.0311	0.1184
DHCer 18	BMI	0.1355	0.0653	0.006 to 0.265	0.2234	0.0411	0.0383	0.1027
HexCer 24:1	BMI	3.325	2.1664	-0.985 to 7.635	0.1671	0.1287	0.0161	0.1483
LacCer 16	BMI	26.9632	17.7917	-8.430 to 62.357	0.1651	0.1335	0.0154	0.1483
LacCer 24:1	BMI	34.0068	19.4368	-4.659 to 72.673	0.1897	0.0839	0.0242	0.1399
CER16/24	BMI	0.0176	0.0222	-0.027 to 0.062	0.0875	0.4289	-0.0045	0.4289
CER18/24	BMI	0.0026	0.0011	0.000 to 0.005	0.2481	0.0229	0.0501	0.1027
CER24:1/24	BMI	0.0211	0.0069	0.007 to 0.035	0.3185	0.0031	0.0905	0.0315
Cer 18	WC	0.2979	0.1511	-0.003 to 0.598	0.2128	0.052	0.0336	0.0881
Cer 24:1	WC	2.1098	0.91	0.299 to 3.920	0.248	0.0229	0.0501	0.0764
DHCer 16	WC	0.1105	0.0541	0.003 to 0.218	0.2201	0.0442	0.0368	0.0881
DHCer 18	WC	0.0739	0.0379	-0.001 to 0.149	0.2106	0.0545	0.0327	0.0881

HexCer 24:1	WC	1.5105	1.2598	-0.996 to 4.017	0.1313	0.234	0.0052	0.26
LacCer 16	WC	18.3813	10.2334	-1.976 to 38.739	0.1946	0.0761	0.0261	0.0952
LacCer 24:1	WC	21.2298	11.2064	-1.063 to 43.523	0.2048	0.0617	0.0302	0.0881
CER16/24	WC	0.0083	0.0129	-0.017 to 0.034	0.0711	0.5203	-0.0071	0.5203
CER18/24	WC	0.0017	0.0007	0.000 to 0.003	0.2696	0.0131	0.0614	0.0657
CER24:1/24	WC	0.012	0.004	0.004 to 0.020	0.3134	0.0037	0.0872	0.037
Cer 18	SBP	0.5125	0.2521	0.011 to 1.014	0.2191	0.0453	0.0364	0.1132
Cer 24:1	SBP	3.7784	1.5134	0.768 to 6.789	0.2658	0.0145	0.0593	0.0879
DHCer 16	SBP	0.1647	0.0909	-0.016 to 0.345	0.1963	0.0735	0.0268	0.147
DHCer 18	SBP	0.0739	0.0642	-0.054 to 0.202	0.126	0.2534	0.0039	0.3621
HexCer 24:1	SBP	3.1769	2.0945	-0.990 to 7.344	0.1652	0.1332	0.0154	0.222
LacCer 16	SBP	40.8145	16.8426	7.309 to 74.320	0.2585	0.0176	0.0554	0.0879
LacCer 24:1	SBP	40.1851	18.6118	3.160 to 77.210	0.2319	0.0338	0.0423	0.1125
CER16/24	SBP	-0.0062	0.0215	-0.049 to 0.037	-0.0316	0.7754	-0.0112	0.7969
CER18/24	SBP	-0.0003	0.0011	-0.003 to 0.002	-0.0285	0.7969	-0.0114	0.7969
CER24:1/24	SBP	0.004	0.0071	-0.010 to 0.018	0.063	0.5689	-0.0082	0.7112

Cer 18	DBP	1.003	0.33	0.346 to 1.659	0.3182	0.0032	0.0903	0.0219
Cer 24:1	DBP	5.2393	2.0345	1.192 to 9.287	0.2735	0.0118	0.0635	0.0394
DHCer 16	DBP	0.2856	0.1208	0.045 to 0.526	0.2526	0.0204	0.0524	0.0511
DHCer 18	DBP	0.2432	0.083	0.078 to 0.408	0.3079	0.0044	0.0837	0.0219
HexCer 24:1	DBP	4.8599	2.8107	-0.731 to 10.451	0.1876	0.0876	0.0234	0.1251
LacCer 16	DBP	48.6216	22.8702	3.125 to 94.118	0.2286	0.0365	0.0407	0.0609
LacCer 24:1	DBP	54.1979	25.0759	4.314 to 104.082	0.2322	0.0336	0.0424	0.0609
CER16/24	DBP	-0.0318	0.0288	-0.089 to 0.025	-0.1211	0.2724	0.0027	0.3405
CER18/24	DBP	0.0001	0.0015	-0.003 to 0.003	0.0094	0.9325	-0.0121	0.9838
CER24:1/24	DBP	0.0002	0.0095	-0.019 to 0.019	0.0022	0.9838	-0.0122	0.9838
Cer 18	HOMA-IR	1.9154	1.2596	-0.590 to 4.421	0.1656	0.1322	0.0156	0.4873
Cer 24:1	HOMA-IR	6.5642	7.7267	-8.807 to 21.935	0.0934	0.3981	-0.0034	0.6925
DHCer 16	HOMA-IR	0.6634	0.4522	-0.236 to 1.563	0.1599	0.1462	0.0137	0.4873
DHCer 18	HOMA-IR	0.7951	0.3078	0.183 to 1.407	0.2743	0.0116	0.064	0.1156
HexCer 24:1	HOMA-IR	-1.227	10.4979	-22.111 to 19.657	-0.0129	0.9072	-0.012	0.9072
LacCer 16	HOMA-IR	51.1062	86.0078	-119.991 to 222.203	0.0655	0.554	-0.0079	0.6925

LacCer 24:1	HOMA-IR	59.8126	94.3573	-127.894 to 247.519	0.0698	0.5279	-0.0073	0.6925
CER16/24	HOMA-IR	-0.0705	0.1062	-0.282 to 0.141	-0.0731	0.5086	-0.0068	0.6925
CER18/24	HOMA-IR	0.005	0.0056	-0.006 to 0.016	0.0984	0.373	-0.0024	0.6925
CER24:1/24	HOMA-IR	0.0162	0.0349	-0.053 to 0.086	0.0512	0.6435	-0.0095	0.7151
Cer 18	HDL-C	-0.3148	0.1967	-0.706 to 0.076	-0.1741	0.1133	0.0185	0.2794
Cer 24:1	HDL-C	-1.9336	1.1947	-4.310 to 0.443	-0.176	0.1094	0.0191	0.2794
DHCer 16	HDL-C	-0.1434	0.0699	-0.282 to -0.004	-0.2211	0.0433	0.0373	0.2165
DHCer 18	HDL-C	-0.1039	0.0487	-0.201 to -0.007	-0.2292	0.036	0.041	0.2165
HexCer 24:1	HDL-C	-0.4604	1.641	-3.725 to 2.804	-0.031	0.7798	-0.0112	0.7798
LacCer 16	HDL-C	-15.3939	13.3711	-41.993 to 11.205	-0.1261	0.253	0.0039	0.3614
LacCer 24:1	HDL-C	-19.4336	14.6351	-48.547 to 9.680	-0.1451	0.1879	0.0091	0.3132
CER16/24	HDL-C	0.0165	0.0166	-0.016 to 0.049	0.1097	0.3206	-0.0	0.4008
CER18/24	HDL-C	-0.0008	0.0009	-0.003 to 0.001	-0.1007	0.3623	-0.0019	0.4025
CER24:1/24	HDL-C	-0.008	0.0054	-0.019 to 0.003	-0.1625	0.1397	0.0145	0.2794
Cer 18	TG	0.0378	0.0612	-0.084 to 0.160	0.0679	0.5392	-0.0075	0.8824
Cer 24:1	TG	-0.0553	0.3728	-0.797 to 0.686	-0.0164	0.8824	-0.0119	0.8824

DHCer 16	TG	0.0131	0.022	-0.031 to 0.057	0.0655	0.5536	-0.0078	0.8824
DHCer 18	TG	0.0185	0.0152	-0.012 to 0.049	0.1325	0.2294	0.0056	0.8824
HexCer 24:1	TG	-0.3455	0.503	-1.346 to 0.655	-0.0756	0.4941	-0.0064	0.8824
LacCer 16	TG	-0.9418	4.1402	-9.178 to 7.294	-0.0251	0.8206	-0.0116	0.8824
LacCer 24:1	TG	-1.9665	4.5397	-10.997 to 7.064	-0.0478	0.666	-0.0099	0.8824
CER16/24	TG	-0.001	0.0051	-0.011 to 0.009	-0.0214	0.8469	-0.0117	0.8824
CER18/24	TG	0.0005	0.0003	-0.000 to 0.001	0.1878	0.0872	0.0235	0.872
CER24:1/24	TG	0.0012	0.0017	-0.002 to 0.005	0.0796	0.4719	-0.0058	0.8824
Cer 18	CRP	3.6466	5.4416	-7.178 to 14.472	0.0738	0.5046	-0.0067	0.8443
Cer 24:1	CRP	-4.0384	33.152	-69.988 to 61.912	-0.0135	0.9033	-0.012	0.9033
DHCer 16	CRP	1.8684	1.9461	-2.003 to 5.740	0.1054	0.3398	-0.0009	0.8443
DHCer 18	CRP	1.9398	1.3506	-0.747 to 4.627	0.1566	0.1547	0.0126	0.7737
HexCer 24:1	CRP	-36.9398	44.6671	-125.797 to 51.917	-0.0909	0.4106	-0.0038	0.8443
LacCer 16	CRP	-140.018	367.9086	-871.905 to 591.869	-0.042	0.7045	-0.0104	0.8443
LacCer 24:1	CRP	-138.6135	403.8103	-941.921 to 664.694	-0.0379	0.7323	-0.0107	0.8443
CER16/24	CRP	-0.2463	0.4541	-1.150 to 0.657	-0.0598	0.5891	-0.0086	0.8443

CER18/24	CRP	0.0378	0.0236	-0.009 to 0.085	0.1741	0.1132	0.0185	0.7737
CER24:1/24	CRP	0.0457	0.1492	-0.251 to 0.343	0.0338	0.7599	-0.011	0.8443

Note: See Table 3 in the main manuscript.
